# The effect of covariate adjustment for baseline severity in acute stroke clinical trials with responder analysis outcomes

**DOI:** 10.1186/1745-6215-14-98

**Published:** 2013-04-11

**Authors:** Kyra M Garofolo, Sharon D Yeatts, Viswanathan Ramakrishnan, Edward C Jauch, Karen C Johnston, Valerie L Durkalski

**Affiliations:** 1 Department of Public Health Sciences, Medical University of South Carolina, 135 Cannon Street, Charleston, SC, 29425, USA; 2 Division of Emergency Medicine, Department of Medicine, Medical University of South Carolina, 169 Ashley Avenue, Charleston, SC, 29425, USA; 3 Department of Neurology, University of Virginia School of Medicine, 81 Hospital Drive, McKim Hall Room 2026, Charlottesville, VA, 22908, USA

**Keywords:** Responder analysis, Sliding dichotomy, Clinical trials, Acute stroke, Modified rankin scale, Baseline severity

## Abstract

**Background:**

Traditionally in acute stroke clinical trials, the primary clinical outcome employed is a dichotomized modified Rankin Scale (mRS). New statistical methods, such as responder analysis, are being used in stroke studies to address the concern that baseline prognostic variables, such as stroke severity, impact the likelihood of a successful outcome. Responder analysis allows the definition of success to vary according to baseline prognostic variables, producing a more clinically relevant insight into the actual effect of investigational treatments. It is unclear whether or not statistical analyses should adjust for prognostic variables when responder analysis is used, as the outcome already takes these prognostic variables into account. This research aims to investigate the effect of covariate adjustment in the responder analysis framework in order to determine the appropriate analytic method.

**Methods:**

Using a current stroke clinical trial and its pilot studies to guide simulation parameters, 1,000 clinical trials were simulated at varying sample sizes under several treatment effects to assess power and type I error. Covariate-adjusted and unadjusted logistic regressions were used to estimate the treatment effect under each scenario. In the case of covariate-adjusted logistic regression, the trichotomized National Institute of Health Stroke Scale (NIHSS) was used in adjustment.

**Results:**

Under various treatment effect settings, the operating characteristics of the unadjusted and adjusted analyses do not substantially differ. Power and type I error are preserved for both the unadjusted and adjusted analyses.

**Conclusions:**

Our results suggest that, under the given treatment effect scenarios, the decision whether or not to adjust for baseline severity when using a responder analysis outcome should be guided by the needs of the study, as type I error rates and power do not appear to vary largely between the methods. These findings are applicable to stroke trials which use the mRS for the primary outcome, but also provide a broader insight into the analysis of binary outcomes that are defined based on baseline prognostic variables.

**Trial registration:**

This research is part of the Stroke Hyperglycemia Insulin Network Effort (SHINE) trial, Identification Number NCT01369069.

## Background

Stroke is a potentially debilitating medical event that affects approximately 800,000 people in the United States each year, leaving as many as 30% of survivors permanently disabled [1]. Given this impact, there is great demand for treatments that significantly improve functional outcome following a stroke. To date, few clinical trials for the treatment of acute stroke have succeeded; of over 125 acute stroke clinical trials, only three successful treatment methods have been identified [2,3].

One of the possible reasons for the excessive number of neutral or unsuccessful stroke trials is the definition of successful outcome utilized in the studies [4]. In clinical trials, stroke outcome is most commonly measured by the modified Rankin Scale (mRS) of global disability at 90 days. The mRS is a valid and reliable measure of functional outcome following a stroke [5]. Past trials have dichotomized mRS scores into “success” and “failure”, scores of 0 to 1 (or 0 to 2) were considered to be “successes” while scores greater than 1 (or 2) were considered to be “failures,” regardless of baseline stroke severity [6-9]. This method fails to take into account the understanding that baseline severity is highly correlated with outcome. New methods, such as the global statistic, shift analysis, permutation testing and responder analysis, are evolving to make better use of the outcome data with the hopes of providing higher sensitivity to detect true treatment effects [2,4,6,9-17].

Responder analysis, also known as the sliding dichotomy, dichotomizes ordinal outcomes into “success” and “failure,” but addresses the drawbacks of traditional dichotomization by allowing the definition of success to vary by baseline prognostic variables. Various trials have implemented the responder analysis where baseline severity is defined by one or many baseline prognostic factors [18-20]. Those study subjects in a less severe prognosis group at baseline must achieve a better outcome to be considered a trial “success,” whereas a less stringent criterion for success is applied to subjects in a more severe baseline prognosis category. The currently enrolling Stroke Hyperglycemia Insulin Network Effort (SHINE) trial employs responder analysis for its primary efficacy outcome [18].

The SHINE trial is a large, multicenter, randomized clinical trial designed to determine the efficacy and safety of targeted glucose control in hyperglycemic acute ischemic stroke patients. While the methodological details of the SHINE trial are discussed elsewhere [18], it should be noted that the primary outcome for efficacy is the baseline severity adjusted 90-day mRS score dichotomized as “success” or “failure” according to a sliding dichotomy. Eligibility criteria for SHINE require that a subject’s baseline NIHSS score must be between 3 and 22, inclusively. Those with a “mild” prognosis, defined by a baseline NIHSS score of 3 to 7, must achieve a 90-day mRS of 0 to be classified as a “success.” Those with a “moderate” prognosis, defined by a baseline NIHSS score of 8 to 14, must achieve a 90-day mRS of 0 to 1 to be classified as a “success.” Finally, those subjects with a “severe” prognosis, defined by a baseline NIHSS score of 15 to 22, must achieve a 90-day mRS of 0 to 2 to be classified as a “success.” By using responder analysis with a trichotomized NIHSS, the threshold for success is stringent for the milder strokes, while the moderate to severe strokes are allowed to have more residual deficits in the threshold for success.

One of the questions that arose from the trial’s Data and Safety Monitoring Board was that of covariate adjustment. Statistical analyses often adjust for prognostic factors, or covariates, that may be predictive of the primary outcome, such as baseline severity [21,22]; however, in the case of SHINE, this prognostic variable is also used to define the outcome. While the literature provides many resources on the design and implementation of responder analysis, as well as examples of trials which used responder analysis, there are no clear resources discussing whether or not statistical analyses should be adjusted for the prognostic variables used to define successful outcome.

This research aims to investigate the effect of covariate adjustment in the responder analysis framework, particularly when the covariate is involved in the definition of successful outcome. The cut-points for the SHINE trial are clinically, rather than statistically, defined and so it is conceivable that adjustment for baseline severity in the statistical analysis may account for additional variation and increase the power to detect a true treatment effect. A simulation study is conducted to assess the operating characteristics (power and type I error) of categorically-adjusted and unadjusted analyses under several possible treatment effect scenarios. In addition, treatment effect estimates and their standard errors are examined across the various scenarios. Since the primary outcome for the SHINE trial is binary, we expect to see an increase of standard error on the treatment effect estimates, consistent with the findings of Robinson and Jewell [23]. However, also consistent with Robinson and Jewell, we expect to see this increase in standard error to be balanced by a movement of the treatment effect estimate away from the null hypothesis.

By examining the effect of covariate adjustment in responder analysis, we aim to define the most appropriate statistical approach to identify true treatment effects. Our findings are not only applicable to the SHINE and other stroke trials which use the mRS for the primary outcome, but also provide insight into the appropriate use of categorical baseline prognostic variables in other trials which use an ordinal scale as a primary outcome measure.

## Methods

Simulation studies were performed to examine the performance of logistic regression models that were unadjusted and adjusted by a trichotomized baseline severity category. Baseline severity category and criteria for successful outcome were defined as in the SHINE trial described above, and are summarized in Table 1. The type I error rate and power were calculated and compared for each method, as were the treatment effect estimates and their standard errors.

**Table 1:** Sliding dichotomy criteria for successful outcome in SHINE trial

**Baseline NIHSS**	**Prognosis group**	**90-day mRS for successful outcome**
3 to 7	Mild	0
8 to 14	Moderate	0, 1
15 to 22	Severe	0, 1, 2

The simulation parameters were guided by the SHINE trial design. A total of 1,000 clinical trials were simulated at sample sizes ranging from 498 to 1,958. This sample size range allowed us to cover the planned SHINE sample size of 1,400 while also examining model behavior at smaller and larger sample sizes. A 1:1 randomization scheme was assumed for the purposes of this investigation. All analyses were performed using SAS version 9.2 (SAS Institute, Cary, NC, USA).

The prevalence of each baseline severity category was guided by data from two prior pilot trials of hyperglycemia management in acute stroke, the Glucose Regulation in Acute Stroke Patients (GRASP) [24] and Treatment of Hyperglycemia in Ischemic Stroke (THIS) [25] pilot trials. In the simulations, 42% of subjects were classified as “mild” at baseline, 32% classified as “moderate”, and the remaining 26% classified as “severe”. This distribution of prognosis categories was imposed using a uniform (0, 1) random variable. In order to simulate 90-day mRS scores for the control group, we examined the distribution of 90-day mRS scores for the control groups in the GRASP and THIS pilot trials. Though the simulation of 90-day mRS scores was primarily driven by the results of the GRASP and THIS pilot trials, the National Institute of Neurological Disorders and Stroke tissue Plasminogen Activator (NINDS tPA) trial control data [26] were used to aid in the approximation of mRS outcome distributions within each of the baseline severity strata. The NINDS tPA control data helped smooth the distribution of mRS scores, as the GRASP and THIS pilot trials each had small sample sizes that resulted in several empty cells after baseline severity stratification. The exact control group distribution of 90-day mRS scores used in the simulation study is shown in Additional file 1: Table S1.

Type I error rates for each method of analysis were obtained by using the same proportion of success for both the control and intervention groups, simulating the null hypothesis of “no treatment effect”. In order to assess the power of each method, a treatment effect was simulated in the data by altering the success prevalence for the intervention group. A 7% treatment effect was used, as this was the minimal clinically relevant absolute difference in favorable outcome between the two treatment groups in the SHINE study plan. For these analyses, power was examined under several scenarios as illustrated in Table 2: (1) a “flat” scenario, in which the 7% treatment effect was held constant over the three baseline severity strata; (2) a “varying” scenario, in which the overall treatment effect is still 7%, but the magnitude within strata is varied, where the mild and moderate groups see the most benefit; (3) another “varying” scenario, in which the severe group sees the most benefit; (4) a “mild harm” scenario, where the mild group sees a harmful treatment effect; and (5) a “severe harm” scenario, in which the severe group sees a harmful treatment effect.

**Table 2:** Success prevalence for simulated treatment effect scenarios

**Baseline severity**	**Treatment effect scenarios**					
	**No treatment effect**	**Flat**	**Varying 1**	**Varying 2**	**Mild harm**	**Severe harm**
**Mild**	25%	32%	33.6%	27%	23%	33%
**Moderate**	35%	42%	44%	44%	50%	48%
**Severe**	15%	22%	17%	27.6%	26.7%	13%

In the first varying scenario, we applied an 8.6% treatment effect in the mild category, a 9% treatment effect in the moderate category and a 2% treatment effect in the severe category; that is, there was an 8.6% increase in prevalence of the 0 mRS for the mild stratum, a 9% increase in the prevalence of the 0 to 1 range of mRS scores for the moderate stratum, and a 2% increase in the prevalence of the 0 to 2 range of mRS scores for the severe stratum. This scenario is relevant to the SHINE trial; it is similar to what we may observe if the investigational treatment is largely beneficial to mild and moderate stroke victims, but only marginally beneficial to victims of severe stroke. The second varying treatment effect scenario applies an opposite effect in which the intensive glucose control intervention is largely beneficial to more severe strokes, but only slightly beneficial to those subjects having mild strokes. Additional file 1: Table S1 shows the exact distribution of 90-day mRS scores for the treatment groups under each of these treatment effect scenarios. These distributions were used to randomly assign 90-day mRS scores to each simulated subject in each simulated trial, with the proportions of success following the scenarios in Table 2. Given a subject’s simulated baseline severity stratum (mild, moderate or severe), an assignment of “success” or “failure” was made according to the sliding dichotomy definitions.

Logistic regression was used to investigate each of these scenarios. The unadjusted case models “success” as a function only of treatment group, while the categorically-adjusted case models “success” as a function of treatment group and severity category. Severity was defined as “mild,” “moderate” or “severe” based on the NIHSS prognosis group discussed in the introduction. Power and type I error rate were based on the proportion of simulated trials at a given sample size which rejected the null hypothesis at a nominal level of 0.05. The treatment effect and its standard error were estimated for each trial.

## Results

**Figure 1:**
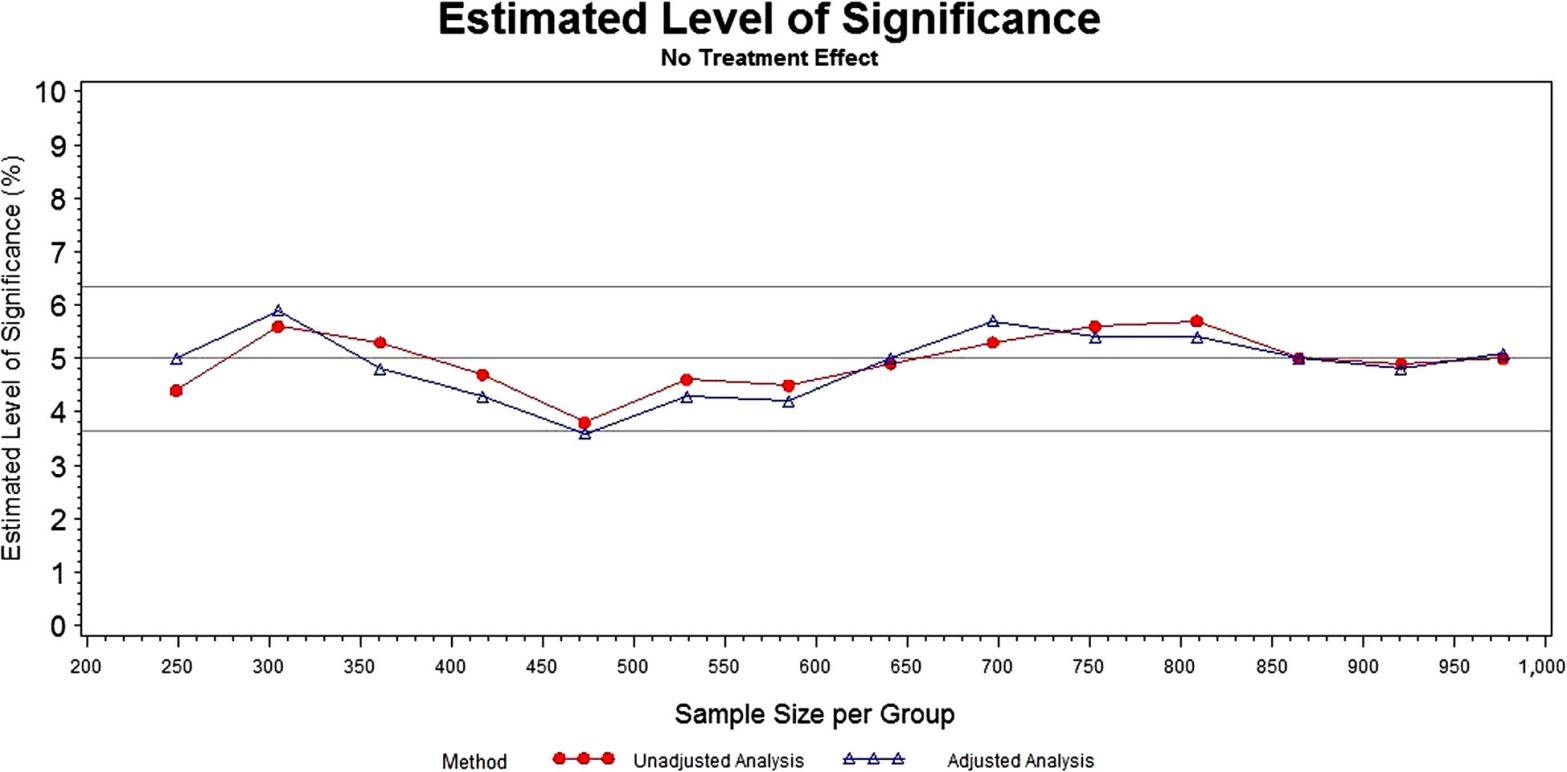
Significance levels of unadjusted and categorically-adjusted methods.

The type I error rate at each sample size for each analysis method is plotted in Figure 1. The nominal 5% reference line is shown, along with the upper and lower 95% confidence limits on this nominal level of significance. The confidence limits were calculated using the formula for binomial proportion 95% confidence intervals. The confidence limits remain the same at each sample size, as they are based on the number of trials at each sample size (1,000) rather than the sample size itself. The type I error rates for both the unadjusted and categorically-adjusted methods are within the 95% confidence limits for all the sample sizes, hovering close to the nominal 5% level.

**Figure 2:**
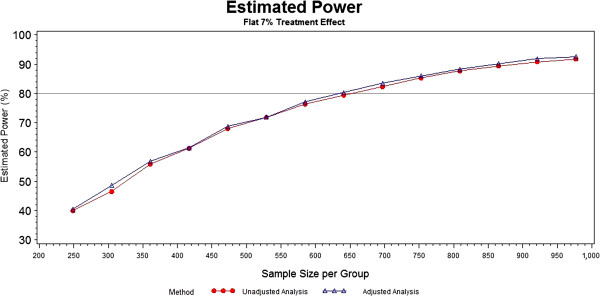
Power of unadjusted and categorically-adjusted methods under a flat 7% treatment effect.

The first investigation of power was under a “flat” treatment effect of 7% where the success rates in the control group were 25%, 35% and 15% in the mild, moderate and severe prognosis groups, respectively. The power estimates for this “flat” treatment effect scenario are plotted in Figure 2. The unadjusted and categorically-adjusted methods do not significantly differ, with the categorically-adjusted method having slightly greater power for most of the sample sizes. As planned by the SHINE study investigators, the 80% power threshold is crossed between 650 and 700 subjects per arm (1,300 to 1,400 subjects total).

**Figure 3:**
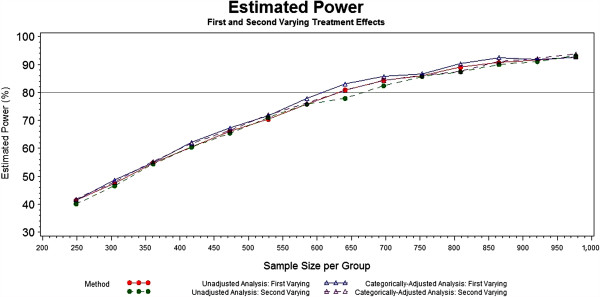
Power of unadjusted and categorically-adjusted methods under the first and second varying 7% treatment effects.

The next two scenarios varied the treatment effects across the mild, moderate and severe baseline categories as 8.6%, 9% and 2%, respectively and 2%, 9% and 12.6%, respectively. The power results for these two scenarios are shown in Figure 3. As in the flat treatment effect scenario, there is no drastic difference in the unadjusted and categorically-adjusted methods with respect to power in these varying treatment effect scenarios.

**Figure 4:**
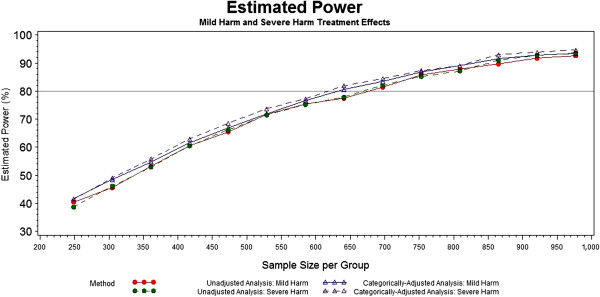
Power of unadjusted and categorically-adjusted methods under mild and severe harm effects.

As previously mentioned, it is conceivable that one of the prognosis groups may experience a slightly harmful treatment effect. When 2% harm is experienced in either the mild or the severe baseline prognosis category, the unadjusted and adjusted analyses still appear to have a similar performance, as shown in Figure 4. In the mild harm scenario, the unadjusted and adjusted power curves are still nearly stacked upon one another, with the power curve for the adjusted analysis pulling slightly above that of the unadjusted analysis at a few points. A more noticeable difference can be seen in the severe harm scenario, where the adjusted analysis consistently has a slightly, though not dramatically, higher power than that of the unadjusted analysis.

**Figure 5:**
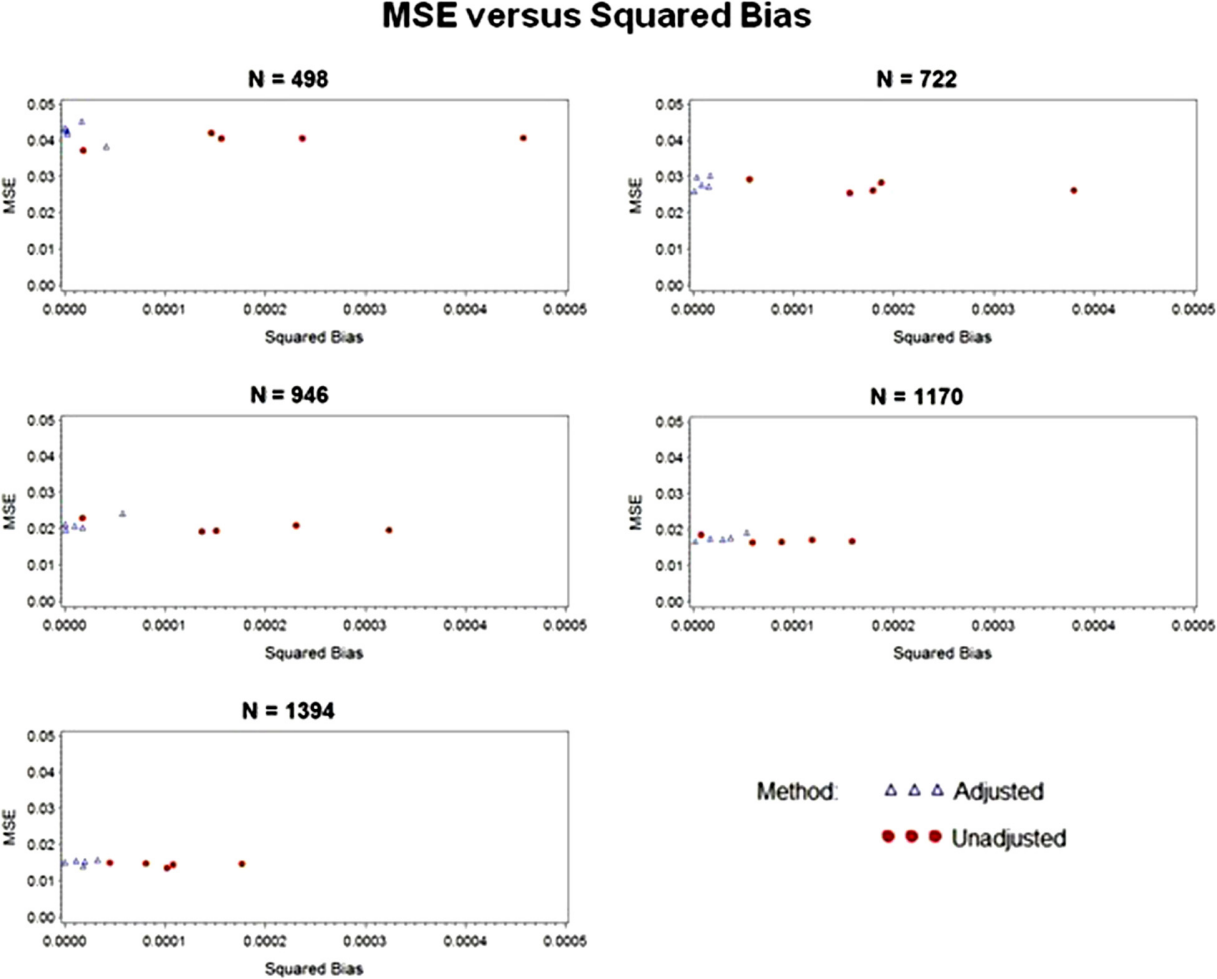
Mean squared error versus squared bias at selected sample sizes.

In addition to the plots in Figures 2, 3 and 4, we also observed the treatment coefficient estimates and their standard errors for the adjusted and unadjusted models under the various treatment effect scenarios at selected sample sizes. The sample sizes of 498, 722, 946, 1,170 and 1,394 were chosen because they are the closest sample sizes to those at which the planned interim and final analyses will take place for SHINE. In addition to model estimates, the true treatment effect coefficient was calculated by pooling the nominal log-odds ratios for each prognosis group. To visualize the bias of the estimate of each treatment effect parameter and their standard errors, the simulation mean squared error (MSE) was plotted against the squared bias in Figure 5. The MSE quantifies the accuracy and precision of an estimate in terms of both the bias (the difference between the true and estimated treatment effect) and the variance of the estimate. By plotting the MSE against the squared bias, we can illustrate the adequacy of the estimator. In Figure 5, the squared bias is depicted on the x-axis and the MSE on the y-axis. While the bias decreases with increasing sample size, the adjusted estimates of the treatment effect parameter are consistently less biased than the unadjusted estimates. For smaller sample sizes, the MSEs for the adjusted analyses are negligibly larger than those for the unadjusted analyses. The treatment coefficients and standard errors are provided in Additional file 2: Table S2.

## Discussion

Successful stroke treatments are desperately needed given stroke’s large and detrimental effect on the worldwide population. Consequently, statistical methods that offer high power to detect a true treatment effect are also needed. With this simulation study, we sought to determine whether adjustment for baseline severity within the responder analysis setting would be beneficial or harmful in terms of power and type I error rates when compared to an unadjusted analysis.

The type I error rates did not differ substantially between the two methods. The experimental type I error rates for both of the methods stayed within the 95% confidence bounds. This is a welcomed result, as a test that is either too liberal or too conservative, (rejects the null hypothesis either more or less than the nominal level, respectively), has implications on the power of the test. The oscillation around the nominal 5% level of significance is likely due to chance, and is to be expected in simulated data. Since neither method shows consistently larger type I error rates than the other, we can conclude that there is no meaningful difference between the two methods with respect to type I error.

The power appears to be approximately the same or slightly higher for the adjusted analyses in the selected scenarios. In the cases where the power is slightly higher, the magnitude is not remarkable and offers little evidence to suggest that adjusting by the single covariate leads to significantly more power. Although the simulation study presented is not exhaustive and, therefore, does not provide additional insight regarding this, the literature by Choi and Hernández suggest that an increase in power could occur as other important prognostic variables are added to the model [27,28]. It is reassuring, however, that neither method appears to be detrimental to power under the given scenarios.

In terms of bias, the unadjusted analyses consistently underestimate the nominal treatment effect, while the adjusted analyses tend to be less biased, but often slightly overestimate the nominal treatment effect. Given the magnitude of the coefficient estimates and their standard errors, neither of these bias tendencies is substantial. In terms of MSE, the two methods do not differ greatly as the sample size increases. At the smaller sample sizes, the adjusted analyses have larger MSE values due to increased standard error; however, as the sample size increases, the MSE values for the two methods converge.

Though negligible differences were identified between the adjusted and unadjusted models, researchers should keep the randomization scheme of the study in mind when deciding whether or not to adjust for baseline severity. In general, it is advisable to “analyze as you randomize,” meaning that any variable used as a stratification variable during randomization should be included as a covariate in the analysis in order to preserve nominal type I error rates and power [22,29]. Baseline severity is often used as a stratification variable in the randomization of acute stroke clinical trials, and should be included as a covariate in these cases.

It is important to note that these analyses adjust categorically for baseline severity. The categories - mild, moderate and severe - are defined by the NIHSS score, which is a larger scale ranging from 0 to 42 (limited to 3 through 22 in SHINE’s inclusion criteria). A one-unit change in the NIHSS cannot easily be interpreted, as this change may have different meanings depending on the combination of neurological impairments and location along the scale. Despite this issue, the NIHSS is sometimes used as a continuous measure in the literature [30,31]. This is not necessarily straightforward and should be done with caution. It is possible that adjusting by the actual NIHSS score will provide additional information to the model and increase or maintain power in some treatment effect scenario(s). However, due to uncertainties in the clinical interpretation of a continuous NIHSS variable, adjustment by actual NIHSS score has been left as a topic for future research.

Adjustment for other baseline prognostic variables may also impact study power under the given scenarios. The inclusion of additional covariates that were not used in defining the primary outcome has not been examined in these scenarios, as it is outside the primary focus of this research. It is conceivable that the addition of multiple covariates could reduce overall power due to the increasing standard error on the treatment effect estimate, as studied by Robinson and Jewell [23] and discussed in the Background section of this paper.

## Conclusions

Our results show negligible differences between analysis methods in the responder analysis setting, suggesting that in most treatment effect scenarios, adjustment for baseline severity in the primary analyses may best be guided by individual study needs rather than a blanket guideline for all studies. Though we have not shown the results here, we did examine other treatment effect scenarios which yield similar results. These scenarios included a flat and varying 15% treatment effect (instead of the 7% specified in the SHINE study plan), as well as a scenario in which the mild group experienced 5% harm.

Overall, these results shed light on the important concept of adjustment in the context of responder analysis. Though this study only examined a single severity scale, its findings are not restricted to use in stroke studies; they can provide insight into the treatment of categorical baseline prognostic covariates in other studies which use responder analysis to define their primary outcome of interest.

## Abbreviations

GRASP: Glucose Regulation in Acute Stroke Patients; mRS: modified Rankin Scale; MSE: mean square error; NIHSS: National Institute of Health Stroke Scale; NINDS tPA: National Institute of Neurological Disorders and Stroke tissue Plasminogen Activator; SHINE trial: Stroke Hyperglycemia Insulin Network Effort trial; THIS: Treatment of Hyperglycemia in Ischemic Stroke.

## Competing interests

The authors declare that they have no competing interests.

## Authors’ contributions

KG carried out the simulation programming and interpretation, manuscript drafting and finalization. SY helped conceive the study concept, assisted in simulation programming and interpretation, and aided in manuscript drafting and finalization. VR helped conceive the study concept, aided in statistical interpretation, as well as manuscript drafting and finalization. KJ and EJ assisted with study concept, design, clinical interpretation, manuscript drafting and finalization. VD helped conceive the study concept, aided in design, analysis and interpretation, as well as manuscript drafting and finalization. VD provided overall supervision as the primary mentor of the first author. All authors read and approved the final manuscript.

## Authors’ information

KG recently completed her master’s degree in biostatistics from the Medical University of South Carolina. This manuscript is a result of her master’s thesis and her work as a graduate student on the SHINE grant. The other authors were her committee members and VD was her primary mentor.

## Supplementary Material

Additional file 1: Table S1 Distribution of 90-day mRS scores. Click here for file

Additional file 2: Table S2 Treatment coefficient estimates and their standard errors for unadjusted and adjusted methods under different treatment effect scenarios. Click here for file
