# Supplementary material for: The effect of covariate adjustment for baseline severity in acute stroke clinical trials with responder analysis outcomes
Source: Trials. 2013 Apr 11;14:98. doi: 10.1186/1745-6215-14-98 (PMC3821551; doi:10.1186/1745-6215-14-98)
Supplement: Additional file 1: Table S1 — Distribution of 90-day mRS scores. [file 1745-6215-14-98-S1.doc]

Supplemental Table 1. Distribution of 90-day mRS Scores

| **Baseline Severity** | ***TREATMENT EFFECT SCENARIO*** | | | | | | |
| --- | --- | --- | --- | --- | --- | --- | --- |
|  | **Control/No Treatment Effect** | | | | | | |
|  | **0** | **1** | **2** | **3** | **4** | **5** | **6** |
| **Mild** | 0.25 | 0.30 | 0.20 | 0.10 | 0.08 | 0.02 | 0.05 |
| **Moderate** | 0.15 | 0.20 | 0.23 | 0.12 | 0.16 | 0.04 | 0.10 |
| **Severe** | 0.03 | 0.05 | 0.07 | 0.19 | 0.20 | 0.21 | 0.25 |
|  | **Flat Treatment Effect** | | | | | | |
| **Mild** | 0.32 | 0.27 | 0.19 | 0.08 | 0.07 | 0.02 | 0.05 |
| **Moderate** | 0.17 | 0.25 | 0.21 | 0.10 | 0.15 | 0.03 | 0.09 |
| **Severe** | 0.04 | 0.06 | 0.12 | 0.18 | 0.18 | 0.19 | 0.23 |
|  | **First Varying Treatment Effect** | | | | | | |
| **Mild** | 0.336 | 0.31 | 0.19 | 0.06 | 0.04 | 0.02 | 0.044 |
| **Moderate** | 0.19 | 0.25 | 0.25 | 0.10 | 0.10 | 0.02 | 0.09 |
| **Severe** | 0.03 | 0.055 | 0.085 | 0.20 | 0.19 | 0.20 | 0.24 |
|  | **Second Varying Treatment Effect** | | | | | | |
| **Mild** | 0.27 | 0.31 | 0.20 | 0.09 | 0.06 | 0.02 | 0.05 |
| **Moderate** | 0.19 | 0.25 | 0.25 | 0.10 | 0.10 | 0.02 | 0.09 |
| **Severe** | 0.04 | 0.09 | 0.146 | 0.18 | 0.12 | 0.194 | 0.23 |
|  | **Mild Harm Treatment Effect** | | | | | | |
| **Mild** | 0.23 | 0.29 | 0.21 | 0.12 | 0.08 | 0.02 | 0.05 |
| **Moderate** | 0.20 | 0.30 | 0.20 | 0.08 | 0.10 | 0.03 | 0.09 |
| **Severe** | 0.05 | 0.09 | 0.127 | 0.13 | 0.16 | 0.20 | 0.243 |
|  | **Severe Harm Treatment Effect** | | | | | | |
| **Mild** | 0.33 | 0.29 | 0.15 | 0.09 | 0.07 | 0.03 | 0.04 |
| **Moderate** | 0.20 | 0.28 | 0.18 | 0.11 | 0.12 | 0.03 | 0.08 |
| **Severe** | 0.02 | 0.04 | 0.07 | 0.20 | 0.21 | 0.21 | 0.25 |
