# Supplementary material for: The effect of covariate adjustment for baseline severity in acute stroke clinical trials with responder analysis outcomes
Source: Trials. 2013 Apr 11;14:98. doi: 10.1186/1745-6215-14-98 (PMC3821551; doi:10.1186/1745-6215-14-98)
Supplement: Additional file 2: Table S2 — Treatment coefficient estimates and their standard errors for unadjusted and adjusted methods under different treatment effect scenarios. [file 1745-6215-14-98-S2.doc]

*Supplemental Table 2: Treatment Coefficient Estimates and Their Standard Errors for Unadjusted and Adjusted Methods Under Different Treatment Effect Scenarios*

| **True Coefficient Values versus Adjusted and Unadjusted Estimates** | | | | | | | | | | | | | | | | | | | | | |
| --- | --- | --- | --- | --- | --- | --- | --- | --- | --- | --- | --- | --- | --- | --- | --- | --- | --- | --- | --- | --- | --- |
| **Scenario** | **True**  **β** | **N=498** | | | | **N=722** | | | | **N=946** | | | | **N=1170** | | | | **N=1394** | | | |
|  |  | **Unadjusted** | | **Adjusted** | | **Unadjusted** | | **Adjusted** | | **Unadjusted** | | **Adjusted** | | **Unadjusted** | | **Adjusted** | | **Unadjusted** | | **Adjusted** | |
|  |  | **β**  **(SSE*)** | **SE_β_**  **(SSE)** | **β**  **(SSE)** | **SE_β_**  **(SSE)** | **β**  **(SSE)** | **SE_β_**  **(SSE)** | **β**  **(SSE)** | **SE_β_**  **(SSE)** | **β**  **(SSE)** | **SE_β_**  **(SSE)** | **β**  **(SSE)** | **SE_β_**  **(SSE)** | **β**  **(SSE)** | **SE_β_**  **(SSE)** | **β**  **(SSE)** | **SE_β_**  **(SSE)** | **β**  **(SSE)** | **SE_β_**  **(SSE)** | **β**  **(SSE)** | **SE_β_**  **(SSE)** |
| **Flat 7%** | 0.3502 | 0.3459  (0.1932) | 0.1992  (0.0048) | 0.3566  (0.1963) | 0.2028  (0.0050) | 0.3427  (0.1713) | 0.1653  (0.0032) | 0.3543  (0.1747) | 0.1681  (0.0034) | 0.3460  (0.1519) | 0.1443  (0.0025) | 0.3578  (0.1558) | 0.1467  (0.0026) | 0.3474  (0.1366) | 0.1296  (0.0020) | 0.3575  (0.1389) | 0.1317  (0.0021) | 0.3435  (0.1227) | 0.1188  (0.0016) | 0.3535  (0.1250) | 0.1206  (0.0017) |
| **Varying 7% (1)** | 0.3551 | 0.3430  (0.2049) | 0.1993  (0.0047) | 0.3598  (0.2131) | 0.2042  (0.0051) | 0.3414  (0.1681) | 0.1654  (0.0032) | 0.3569  (0.1730) | 0.1692  (0.0034) | 0.3399  (0.1442) | 0.1444  (0.0024) | 0.3545  (0.1459) | 0.1476  (0.0025) | 0.3442  (0.1309) | 0.1297  (0.0019) | 0.3592  (0.1324) | 0.1326  (0.0020) | 0.3450  (0.1164) | 0.1188  (0.0016) | 0.3594  (0.1188) | 0.1214  (0.0017) |
| **Varying 7% (2)** | 0.3500 | 0.3375  (0.2012) | 0.1990  (0.0045) | 0.3485  (0.2051) | 0.2027  (0.0048) | 0.3375  (0.1594) | 0.1651  (0.0031) | 0.3489  (0.1618) | 0.1679  (0.0033) | 0.3383  (0.1385) | 0.1442  (0.0023) | 0.3489  (0.1406) | 0.1465  (0.0025) | 0.3423  (0.1284) | 0.1296  (0.0019) | 0.3514  (0.1298) | 0.1316  (0.0020) | 0.3410  (0.1218) | 0.1188  (0.0016) | 0.3501  (0.1237) | 0.1206  (0.0017) |
| **Mild Harm** | 0.3520 | 0.3366  (0.2012) | 0.1990  (0.0045) | 0.3533  (0.2070) | 0.2044  (0.0050) | 0.3386  (0.1615) | 0.1651  (0.0031) | 0.3559  (0.1656) | 0.1693  (0.0034) | 0.3397  (0.1391) | 0.1442  (0.0023) | 0.3562  (0.1428) | 0.1477  (0.0026) | 0.3426  (0.1287) | 0.1296  (0.0019) | 0.3574  (0.1316) | 0.1327  (0.0021) | 0.3416  (0.1202) | 0.1187  (0.0016) | 0.3565  (0.1238) | 0.1216  (0.0018) |
| **Severe Harm** | 0.3558 | 0.3344  (0.2008) | 0.1991  (0.0045) | 0.3557  (0.2085) | 0.2056  (0.0050) | 0.3363  (0.1611) | 0.1651  (0.0031) | 0.3587  (0.1667) | 0.1703  (0.0034) | 0.3378  (0.1392) | 0.1442  (0.0023) | 0.3589  (0.1448) | 0.1485  (0.0026) | 0.3432  (0.1293) | 0.1296  (0.0019) | 0.3619  (0.1335) | 0.1334  (0.0021) | 0.3425  (0.1208) | 0.1187  (0.0016) | 0.3615  (0.1256) | 0.1222  (0.0018) |

*SSE represents simulation standard error.
